# Digital registration versus cone-beam computed tomography for evaluating implant position: a prospective cohort study

**DOI:** 10.1186/s12903-024-04088-x

**Published:** 2024-03-04

**Authors:** Xinrui Han, Donghao Wei, Xi Jiang, Ping Di, Chun Yi, Ye Lin

**Affiliations:** Department of Oral Implantology, Peking University School and Hospital of Stomatology & National Clinical Research Center for Oral Diseases & National Engineering Research Center of Oral Biomaterials and Digital Medical Devices & Beijing Key Laboratory of Digital Stomatology, 22 Zhongguancun South Avenue, Haidian District, Beijing, 100081 PR China

**Keywords:** Dental implant, Accuracy, Digital registration, Cone-beam computed tomography, Intraoral scan

## Abstract

**Background:**

Postoperative cone-beam computed tomography (CBCT) examination is considered a reliable method for clinicians to assess the positions of implants. Nevertheless, CBCT has drawbacks involving radiation exposure and high costs. Moreover, the image quality can be affected by artifacts. Recently, some literature has mentioned a digital registration method (DRM) as an alternative to CBCT for evaluating implant positions. The aim of this clinical study was to verify the accuracy of the DRM compared to CBCT scans in postoperative implant positioning.

**Materials and methods:**

A total of 36 patients who received anterior maxillary implants were included in this clinical study, involving a total of 48 implants. The study included 24 patients in the single implant group and 12 patients in the dual implant group. The postoperative three-dimensional (3D) positions of implants were obtained using both CBCT and DRM. The DRM included three main steps. Firstly, the postoperative 3D data of the dentition and intraoral scan body (ISB) was obtained through the intraoral scan (IOS). Secondly, a virtual model named registration unit which comprised an implant replica and a matching ISB was created with the help of a lab scanner and reverse engineering software. Thirdly, by superimposing the registration unit and IOS data, the postoperative position of the implant was determined. The accuracy of DRM was evaluated by calculating the Root Mean Square (RMS) values after superimposing the implant positions obtained from DRM with those from postoperative CBCT. The accuracy of DRM was compared between the single implant group and the dual implant group using independent sample t-tests. The superimposition deviations of CBCT and IOS were also evaluated.

**Results:**

The overall mean RMS was 0.29 ± 0.05 mm. The mean RMS was 0.30 ± 0.03 mm in the single implant group and 0.29 ± 0.06 mm in the dual implant group, with no significant difference (*p* = 0.27). The overall registration accuracy of the IOS and CBCT data ranged from 0.14 ± 0.05 mm to 0.21 ± 0.08 mm.

**Conclusion:**

In comparison with the 3D implant positions obtained by CBCT, the implant positions located by the DRM showed clinically acceptable deviation ranges. This method can be used in single and dual implant treatments to assess the implant positions.

## Introduction

Implant-supported restorations are widely recognized as a reliable and predictable solution to replacement of missing teeth. The three-dimensional (3D) positions of implants are crucial for the long-term prognosis of implant restorations [1]. Radiographic examination is the commonly accepted method for clinicians to acquire the implant positions in the treatment process [2], with the aim of assessing the optimal 3D position of the implant, damages to the important surrounding anatomical structures [3], and the accuracy of the surgical guide [4].

Currently, the radiographic examinations used for postoperative evaluation of dental implant placement include periapical radiographs, panoramic radiographs and cone beam computed tomography (CBCT) [5]. Nonetheless, radiographic examinations impose additional biological and economic burdens on patients. In recent years, there has been a growing public concern regarding radiation exposure associated with imaging examinations [6]. Periapical radiography has a radiation dose of less than 2μSv, panoramic radiography ranges from 3 to 24μSv [5], and CBCT ranges from 28 to 652μSv [7]. Meanwhile, the image quality of all the three examinations can be affected by patient movement and metal artifacts [8]. Periapical and panoramic radiographs provide only two-dimensional (2D) information, and their images are subject to distortion and magnification. Standardization for intraoral radiographies were proposed by Cosola et al. in order that they can be used in more precise way [9]. CBCT is the commonly used imaging examination that allows for 3D visualization of implant positioning.

Recently, several studies have introduced a digital registration method (DRM) that utilizes intraoral scan (IOS) and registration software to acquire the 3D position of implants [4, 10–13]. The DRM procedure begins with intraoral insertion of an intraoral scan body (ISB) to acquire a digital model of both the ISB and the dentition through IOS. Next, the ISB is connected with the standard implant replica and scanned using a high-precision model scanner. Subsequently, the digitized implant is aligned with the IOS digital model through the ISB portion in the reverse engineering software via registration process. This is followed by aligning the pre-operative CBCT data with the intraoral scan data based on tooth surfaces. This method provides an alternative to postoperative CBCT for evaluating the 3D implant position and its spatial relationship with adjacent anatomical structures [14].

In previous in vitro studies, the implant positions obtained by the DRM had a high degree of spatial overlap with the implant positions located with CBCT after being aligned in the same coordinate system [4, 10]. An in vitro study designed by Zhou et al. [10] compared the virtual implant obtained by the DRM with the implant visualized by CBCT, indicating that the average linear deviation between the two implants was less than 0.3 mm, while the angular deviation was less than 0.8°, which was considered clinically acceptable. Zhou et al. [10] recommended that further clinical studies were necessary to verify the feasibility and accuracy of the DRM in clinical practice. In another in vitro study conducted by Yi et al. [4], the DRM and CBCT method exhibited a high level of agreement in evaluating the accuracy of implant positioning. The study involved 40 resin models under controlled conditions, devoid of salivary or blood interference. Consequently, the author emphasized the need for further clinical studies to validate the efficacy of this novel approach.

In previous clinical studies, the DRM was used to obtain 3D position of implants for evaluating accuracy of the surgical guide [11, 15–17]. In these studies, the implant position obtained by this method was directly regarded as the actual implant position without the comparison or verification against postoperative CBCT scans. Additionally, these clinical studies did not investigate whether the accuracy of this digital method was affected by the number of implants. Derksen et al. [15] conducted a clinical study using the DRM to verify the accuracy of guided implant surgery. Postoperative IOS was performed after connecting an ISB to the inserted implant. The Standard Tessellation Language (STL) files of the IOS models were imported into a dedicated software and the virtual implant was calculated and visualized. The 3D discrepancies between the planned and actual implant positions were then assessed to validate the accuracy of surgical guides. The authors suggested that the digital method could be used for implant positioning. However, due to the small sample size of the study (only 6 implants from 3 patients), the authors suggested the necessity for larger sample clinical studies to verify the accuracy of the DRM compared to CBCT.

Therefore, the purpose of this prospective cohort study was to evaluate the accuracy of the DRM for implant positioning and compare it with the CBCT-based method, as well as to investigate the differences in the accuracy of acquiring 3D positions for single and dual implants using the DRM.

## Material and methods

### Patient enrollment protocol

This study recruited patients from the Peking University School and Hospital of Stomatology between April 2020 and October 2022. The study received ethical approval from the Institutional Review Board of Peking University School and Hospital of Stomatology (Approval Number: PKUSSIRB-201839133). All patients were provided with detailed information regarding the study protocol, and written consent was obtained from each participant.

#### Inclusion criteria

Single or dual missing maxillary incisors that required implant restoration.

#### Exclusion criteria

① local or systemic contraindications for implant therapy; ② uncontrolled periodontitis or with teeth exhibiting mobility levels more than I°; ③ existence of metal restorations or implant prostheses.

### Sample size calculation

A previous in vitro study reported by Yi C. et al. [4] evaluated the accuracy of guided implant surgery via the DRM and CBCT scans. Based on the results of the study, the result of interclass correlation coefficients (ICCs) were used for sample size calculation. In PASS software (version 15; NCSS, LLC., Kaysville, Utah, USA), a sample size of 36 was calculated to be necessary to achieve a power of 90% (β = 0.10) for detecting an ICC of 0.90 under the alternative hypothesis, assuming that the ICC under the null hypothesis was 0.75 and the significance level was 0.05 (α = 0.05).

### Treatment procedures and data collection

Based on the number of missing teeth in the patients, the patients were divided into two groups: the single implant group (24 patients) and the dual implant group (12 patients). Each implant site in the single implant group as well as in the dual implant group was numbered from 1 to 24, respectively. In this study, the postoperative CBCT data and the IOS data of the enrolled patients were used. CBCT scans were essential radiographic examinations before and after implant surgery in the maxillary anterior teeth region to assess the preoperative horizontal bone volume, postoperative bucco-palatal position of the implants, and the outcomes of bone grafts. These CBCT scans were not scanned for research purposes and therefore did not subject patients to additional radiation doses. IOS data were obtained specifically for study purposes.

**Fig. 1:**
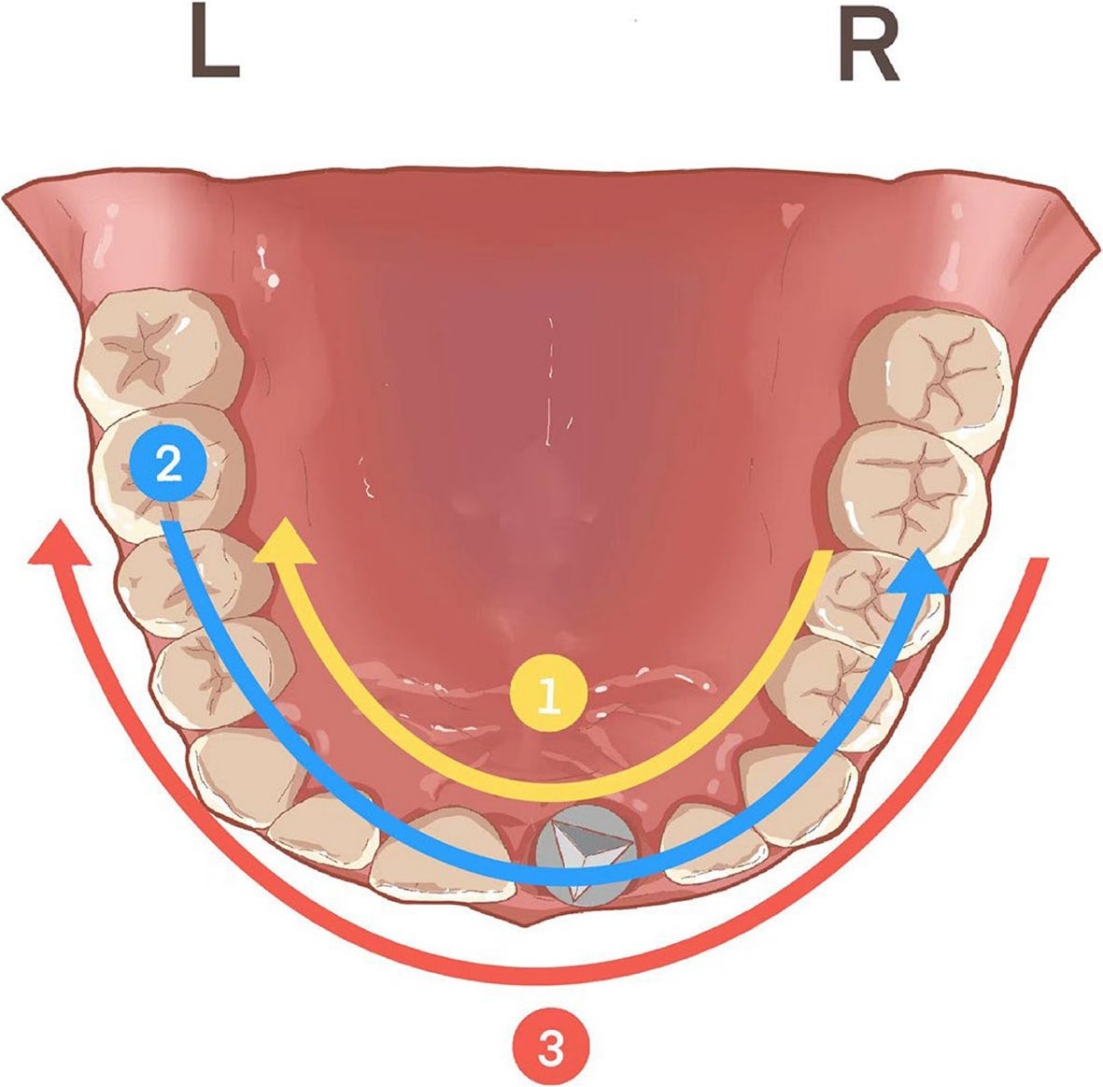
Graphic illustration of the IOS pattern

**Fig. 2:**
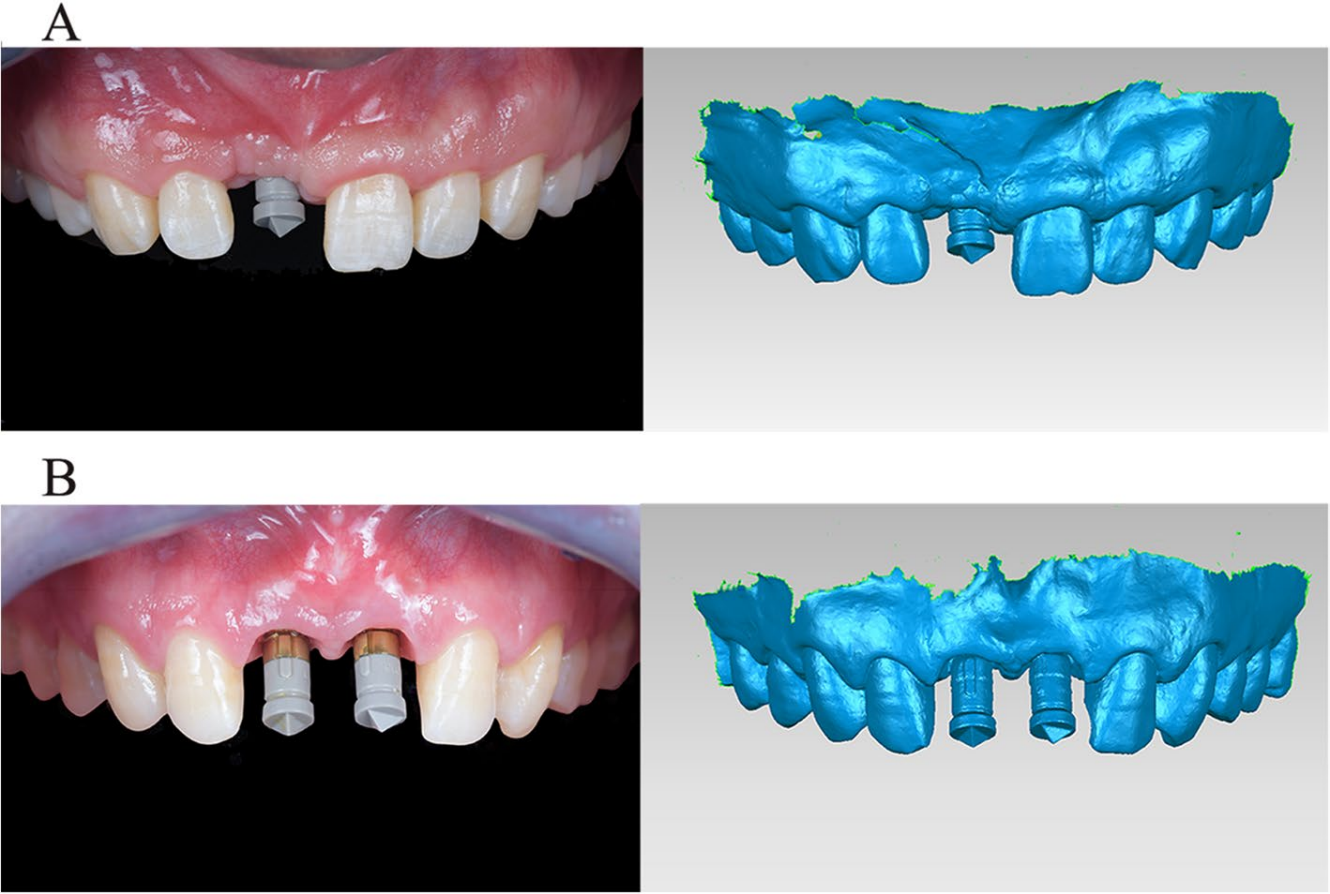
Before restorations, scan bodies were positioned and IOS were performed. **A** single implant group;** B** dual implant group. (STL-IOS)

The patients were received implant placement (Camlog Screw-Line Implant, Camlog Biotechnologies AG, Basel, Switzerland) with or without simultaneous bone augmentation. CBCT scans (Planmeca ProMax 3D; Planmeca Oy, Finland) were taken immediately after implant surgery to check the 3D position of the inserted implants and the outcomes of bone grafts. The exposure parameters of the CBCT scans were set as follows: field-of-view (FOV) diameter, 13 × 10cm; FOV height, 5.6cm; acceleration voltage, 90kV; beam currency, 8.0mA; and voxel size, 0.2mm. After CBCT scans, shadows and streaks caused by implants were removed in the software (Planmeca Romexis; Planmeca Oy, Finland). After a healing period of 4 to 6 months, conventional impressions were taken to fabricate the interim or permanent restorations. Before impression taking, the ISB of the implants were inserted and IOS of the upper dentition were obtained using an optical scanner (3Shape TRIOS Color; 3Shape, Copenhagen, Denmark). All IOS data were acquired by the same experienced operator using the following scanning strategy (Fig. 1): started on lingual surfaces of second premolar on patient’s right side and continued to lingual surfaces of contralateral second premolar. The next sequence captured the occlusal surfaces back to the starting premolar. The next sequence was buccal surfaces from the starting premolar to the buccal surfaces of contralateral second premolar. For further analysis, all CBCT data were exported as Digital Imaging and Communications in Medicine (DICOM) format. All IOS data were exported as STL format named as “STL-IOS” (Fig. 2).

### Two methods to evaluate the implant positions

#### The DRM

**Fig. 3:**
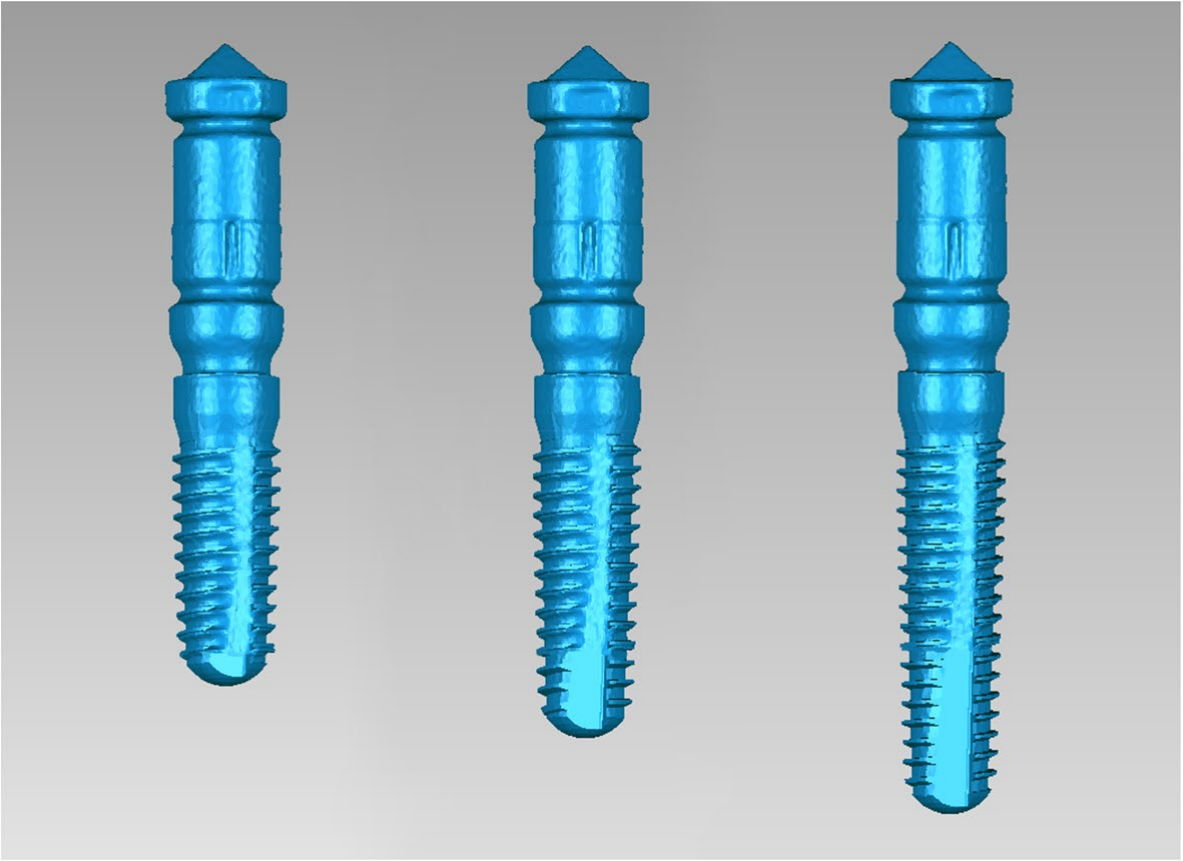
ISBs were positioned in vitro on the three length of 3.8mm Camlog implants and scanned by a laboratory scanner (from left to right, 3.8 mm × 11 mm, 3.8 mm × 13 mm and 3.8 mm × 16 mm). (STL-Registration unit)

In this study, dental implants with dimensions of 3.8 × 11 mm, 3.8 × 13 mm, and 3.8 × 16 mm were placed. The DRM involved three steps. Firstly, the “STL-IOS” data of each patient was obtained. Secondly, the three different lengths of 3.8 mm diameter Camlog implants were connected to matching ISB respectively in vitro. Since the relationship between the implant and ISB was fixed, the 3D position of the postsurgical implant could be represented by the ISB. The combined structure (implant and ISB) constituted a registration unit. Subsequently, the three types of registration units were scanned using a laboratory scanner (3Shape E4; 3Shape, Copenhagen, Denmark) and the data were exported as STL files and named as “STL-Registration unit” (Fig. 3). Thirdly, the “STL-IOS” was registered with the corresponding “STL-Registration unit” based on the ISB via best-fit algorithm in a reverse engineering software (Geomagic Studio 2014; Geomagic, 3D Systems, Rock Hill, SC, USA).

**Fig. 4:**
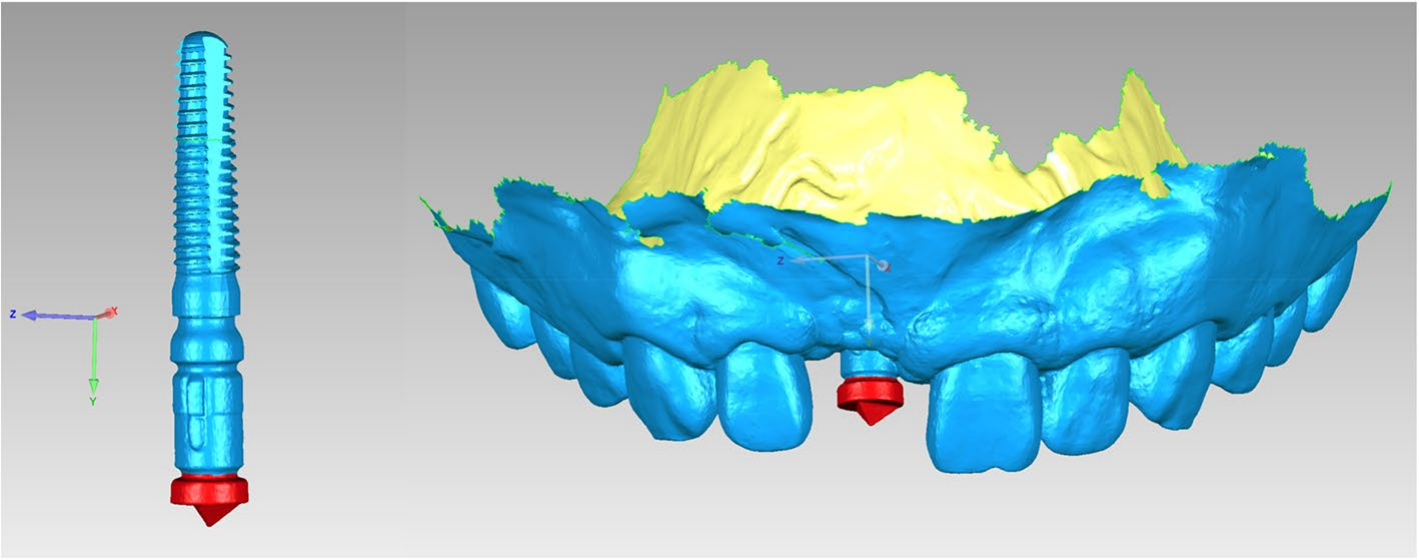
The common structure within both STL files were selected (in red color) for registration

The ISB served as the registration target in the best-fit alignment procedure owing to its common structure within both STL files (“STL-IOS” and “STL-Registration unit”). Due to variations in implant depth and gingival thickness among patients, the portion of the ISB exposed within the oral cavity also differed significantly. Therefore, only the top feature region (the triangular and short cylindrical parts) (Fig. 4) of the ISB was selected for the alignment procedure.

The best-fit algorithm is the most commonly used algorithm for aligning two irregular and complex surface digitized models [18–21]. It utilizes an iterative closest point (ICP) algorithm to align two meshes, minimizing the discrepancy between the two point clouds by iteratively adjusting the transformation to minimize an error metric.

**Fig. 5:**
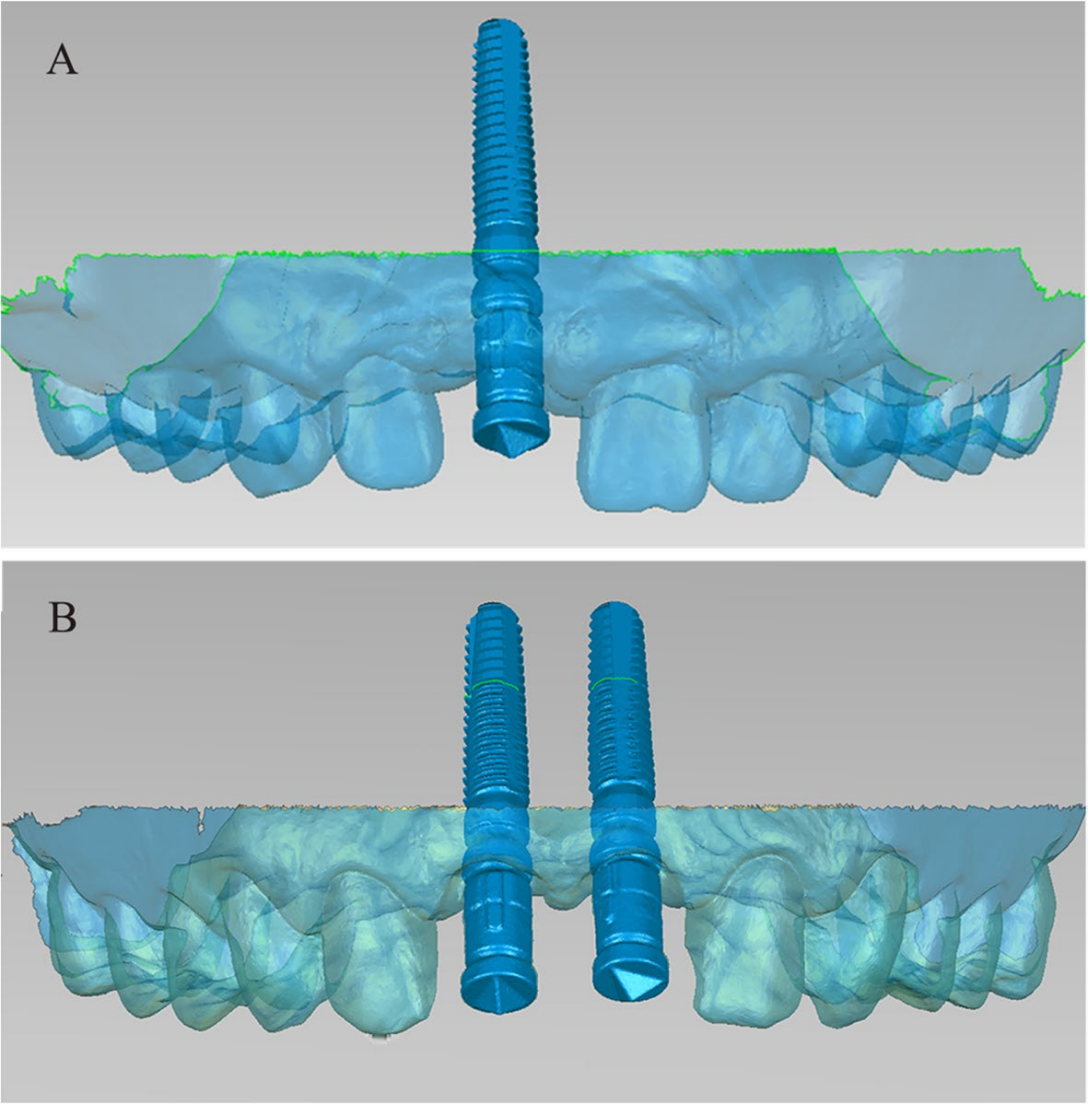
The IOS models were aligned with the corresponding types of registration unit based on the structure of the scan bodies. **A** single implant group; **B** dual implant group. (STL-IOS with implant)

The alignment procedure was carried out using the reverse engineering software (Geomagic Studio 2014; Geomagic, 3D Systems, Rock Hill, SC, USA). The “STL-IOS” and “STL-Registration unit” files were imported into the software. The “STL-IOS” model was set as the reference model, while the “STL-Registration unit” model was set as test model for registration process. After selecting the top feature region of the ISB as the registration target in both models, the “STL-Registration unit” were aligned with the “STL-IOS” via the best-fit alignment algorithm by the software, which aligned both meshes with the shortest distance between every data point. The two aligned digital models were merged into a single digital model named as “STL-IOS with implant” (Fig. 5). Then, the postsurgical implant position was obtained using the DRM.

The mean root mean square (mRMS) error was computed to assess the discrepancy between the two aligned models. The Geomagic Studio 2014 software (Geomagic, 3D Systems, Rock Hill, SC, USA) automatically computed the mRMS value to determine the alignment error. Additionally, the overall 3D deviations for each pair of aligned models could be visually observed through a color spectrum.

#### The CBCT scans

**Fig. 6:**
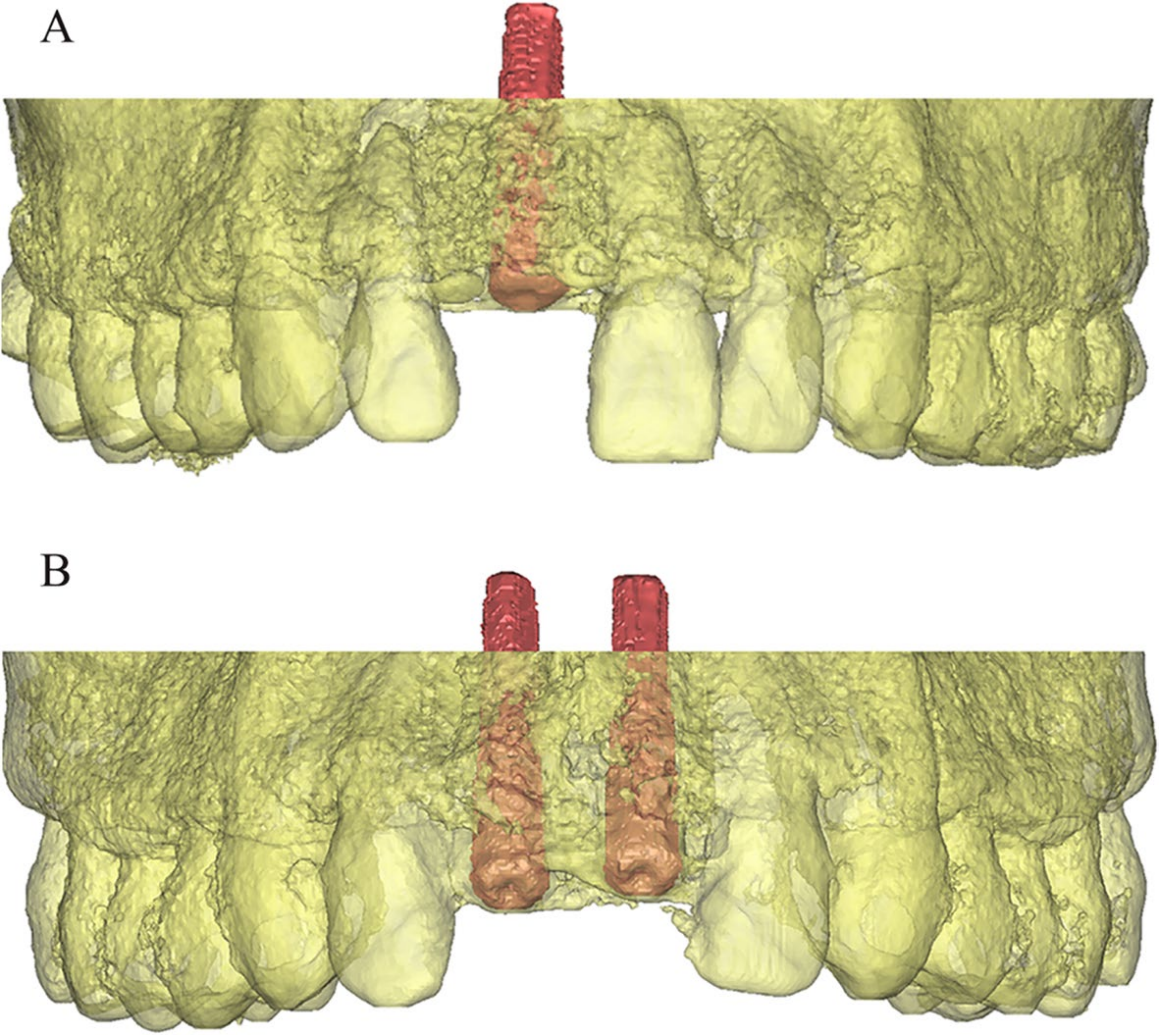
The post-surgical CBCT images. The teeth and alveolar bone were displayed in yellow, while the implants were distinguished by being highlighted in red. **A** single implant group; **B** dual implant group. (STL-CBCT)

For each patient, postoperative CBCT data (DICOM format) were transferred to volumetric imaging software (Mimics 15.0; Materialise, Leuven, Belgium). The 3D masks of the maxilla (including the teeth and alveolar bone) and the inserted implant were extracted separately via threshold segmentation based on their Hounsfield unit values. The models reconstructed from the CBCT scans were saved in STL format, which named as STL-CBCT (Fig. 6).

### The 3D comparison of the implant positions acquired by CBCT and the DRM

For each patient, the “STL-IOS with implant” models were imported into a dedicated analysis software (ProPlan CMF; Materialise, Leuven, Belgium) and registered with the “STL-CBCT” models based on the tooth surfaces from the right second premolar to the left second premolar via multi-point registration.

**Fig. 7:**
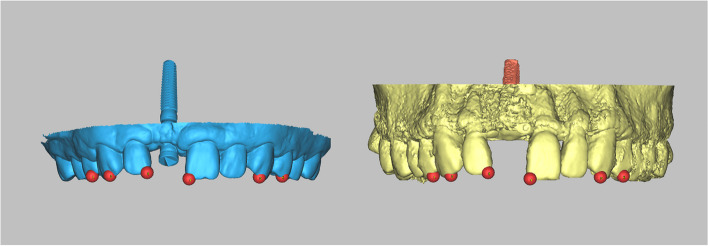
The multi-point registration approach was employed to register the “STL-IOS with implant” with the “STL-CBCT”

Given the different data types of CBCT and IOS (CBCT being volume data and IOS being surface data), we adopted a multi-point registration approach for superior registration results. This approach involved the initial manually selection of multiple points for preliminary registration, followed by a secondary registration using the best-fit algorithm by the software to achieve the final result. Therefore, from the perspective of registration principles, the algorithm behind multipoint registration is also based on the best-fit registration algorithm. The initial multi-point registration aimed to roughly align the coordinates of the two complex 3D surface models, assist the software in identifying the registration area, reduce computational complexity, and obtain a more ideal registration result. This approach has been widely used in the previous literature for aligning the CBCT and IOS data [22–26]. Specifically, for the initial registration process, six to eight points were manually selected on the cusp tips of the teeth on the CBCT scan as well as on the IOS in our study (Fig. 7). The Proplan software (ProPlan CMF; Materialise, Leuven, Belgium) automatically performed a secondary registration, adjusting the preliminary registration results to obtain a more optimal outcome.

**Fig. 8:**
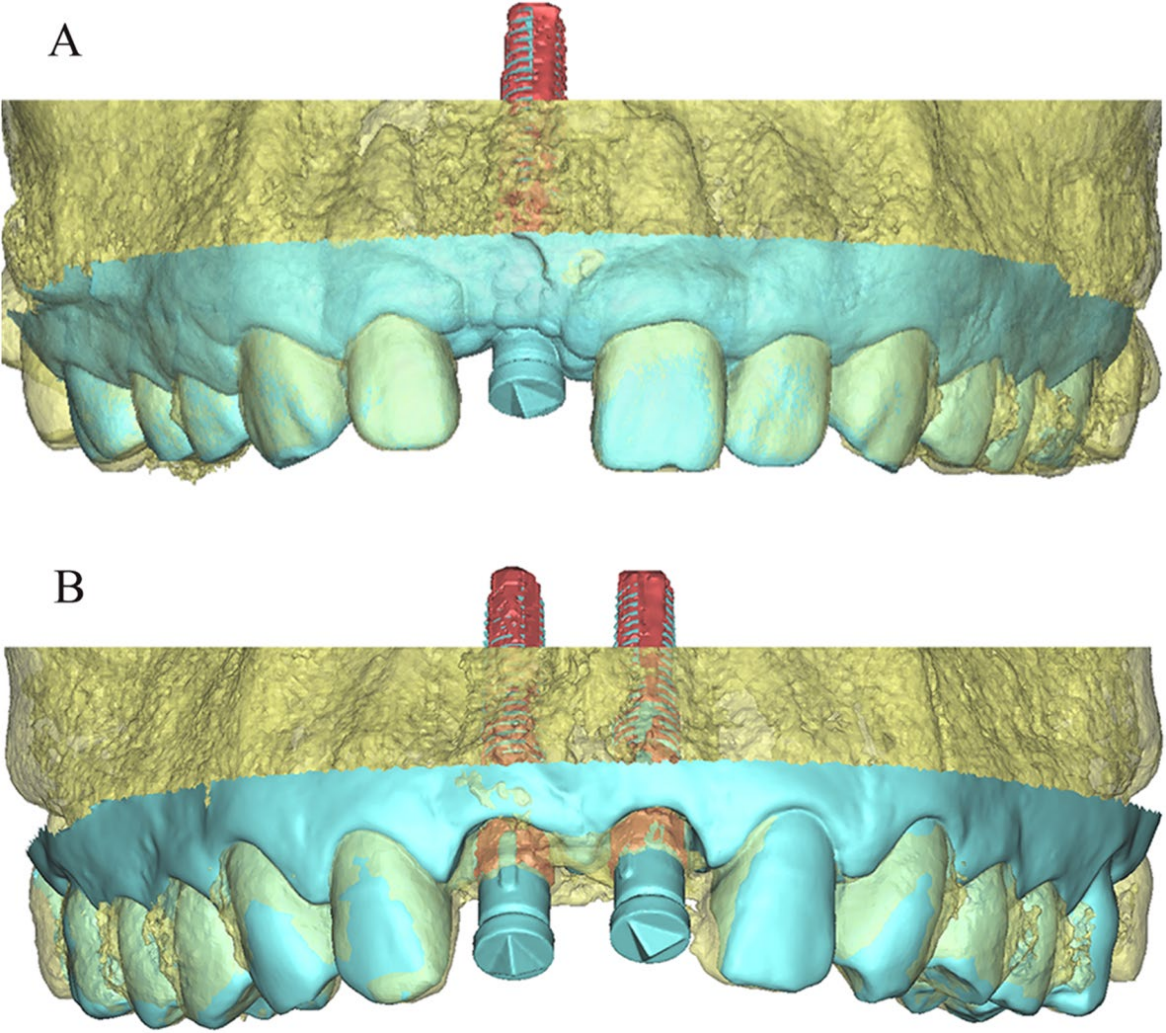
“STL-IOS with implant” was aligned with “STL-CBCT” based on the tooth surfaces (from the right second premolar to the left second premolar). **A** single implant group;** B** dual implant group

The superimposed “STL-IOS with implant” and “STL-CBCT” were exported as a new STL file (Fig. 8) and imported to the reverse engineering software (Geomagic Studio 2014) for further analysis.

Before evaluation the accuracy of the DRM and CBCT in 3D implant positioning, we quantitatively measured the deviations on the natural teeth between IOS and CBCT data in Geomagic software (Geomagic Studio 2014) in order to validate the accuracy of the registration process in this study. Four adjacent teeth, including those proximal and distal to the implants, were selected for measurements on their 2D cross-sections determined by the long axis and the gingival zenith. On each cross-sectional plane, three measurement points, namely the midpoint of incisal edge, labial prominence and apex of the lingual tubercle, were selected for deviation analysis. For each patient, a total of 12 measurement points were identified on the four adjacent tooth surfaces, and the deviation values were measured.

**Fig. 9:**
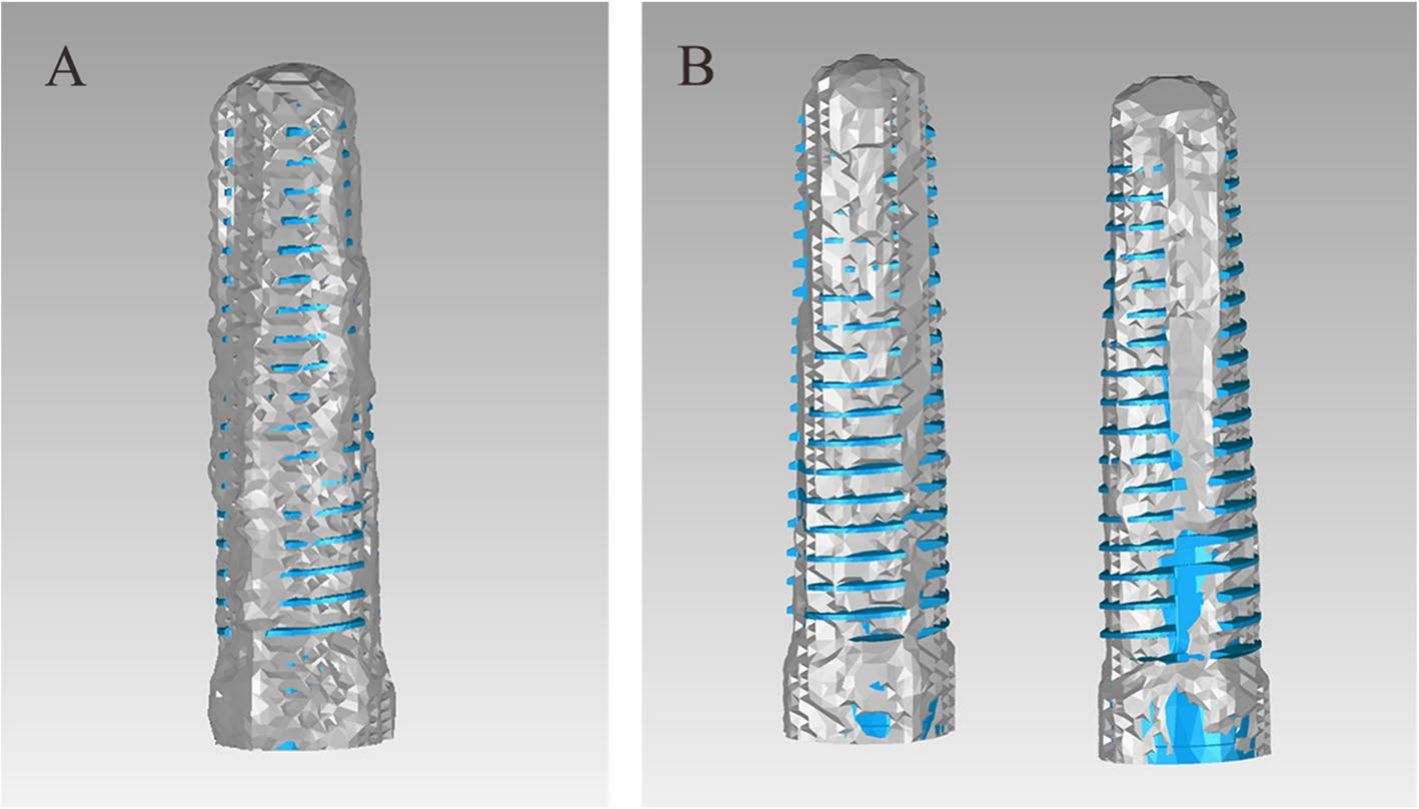
The two superimposed implants obtained by two methods were selected in the reverse engineering software for visual analysis. The gray-colored implants represented those obtained by CBCT scans, and the blue-colored implants represented those obtained by the DRM. **A** single implant group; **B** dual implant group

The superimposed digital model was then trimmed to remove the teeth, gingiva, and alveolar bone, leaving only the area of the implants obtained by the above two methods (Fig. 9). Root Mean Square (RMS) was used as the main parameter to evaluate the 3D deviation between the 3D positions of the implants obtained by the two methods. The average values of linear deviation on the four teeth were calculated.

**Fig. 10:**
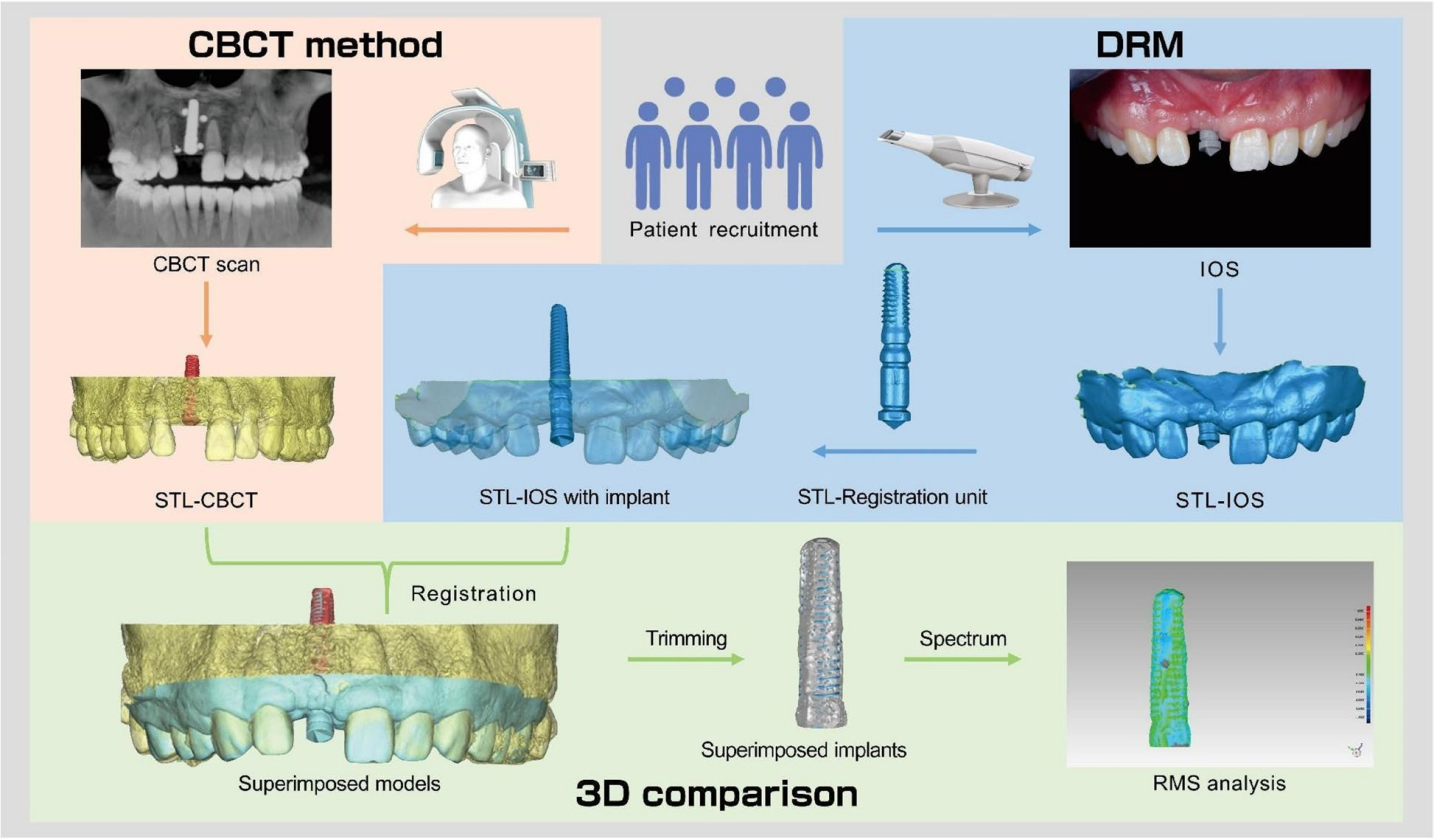
The primary workflow of this study comprised three stages, in which respective implant positions were obtained through CBCT and DRM. Then, the 3D comparison was performed to evaluate the accuracy of DRM

The main workflow of the study was depicted in Fig. 10.

### Statistical analysis

Statistical analysis was performed using SPSS software (version 27.0; IBM Corp., Armonk, NY, USA) at a significance level of p = 0.05. Descriptive statistics were generated and the Kolmogorov–Smirnov test (α = 0.1) was performed for all parameters. All data were normally distributed. Descriptive statistical analysis was performed for all parameters. Independent sample t-test was used to compare the results between the two groups.

## Result

Thirty-six patients finished the implant treatment, CBCT scans and IOS.

**Fig. 11:**
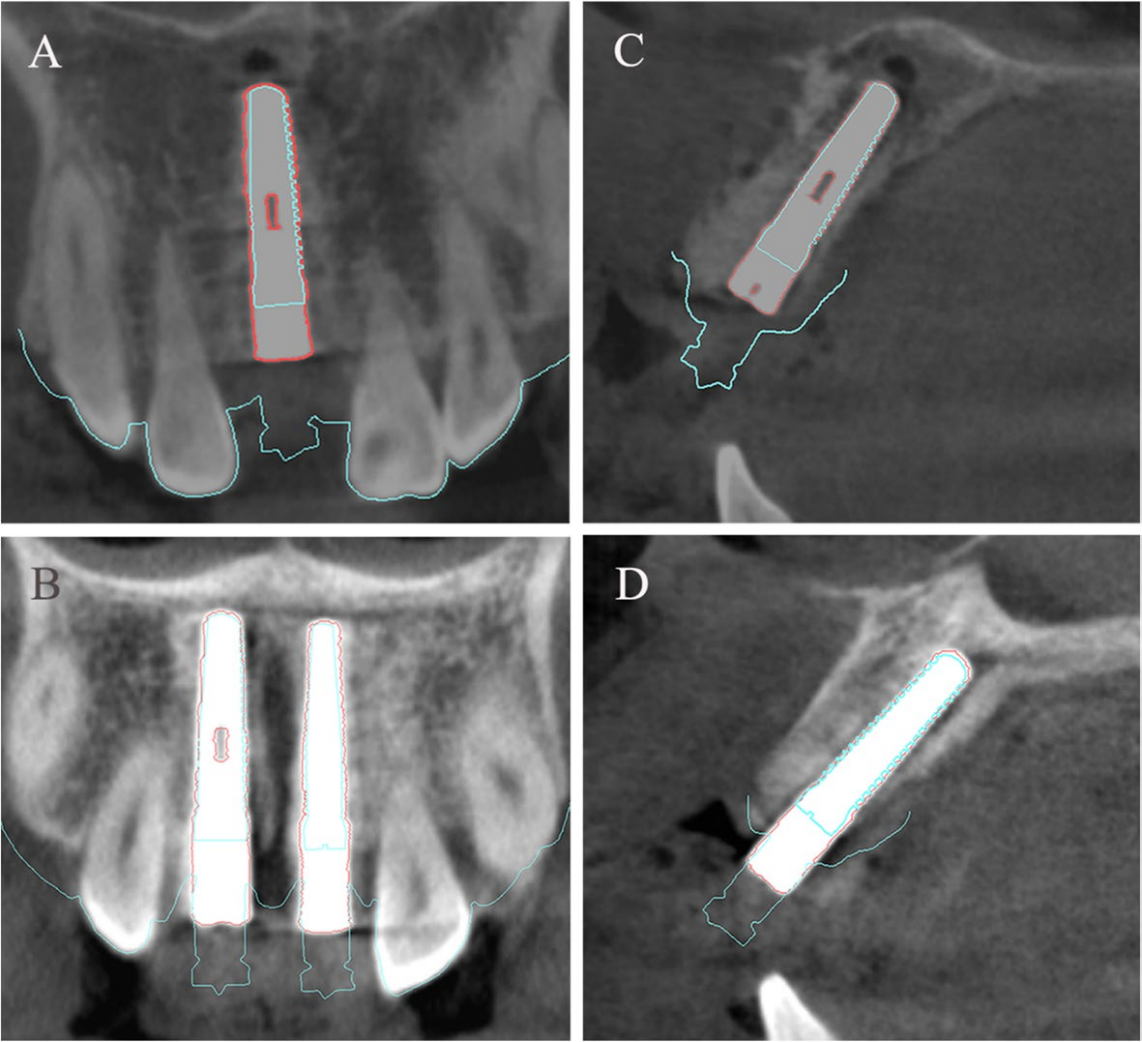
The coronal (**A** and **B**) and sagittal (**C** and **D**) cross-sectional planes alongside the axis of the implants. The blue lines delineated the implant positions obtained by IOS, while the red lines depicted the implant positions obtained by CBCT scans. **A** and** C** single implant group;** B** and** D** dual implant group

The 2D cross-sectional images in the coronal and sagittal planes depicted the implant positions obtained from IOS with those obtained from CBCT (Fig. 11). It was visually evident that the registration performance in the dentition was satisfactory, with a high degree of consistency between the CBCT and DRM in determining the implant positions.

**Fig. 12:**
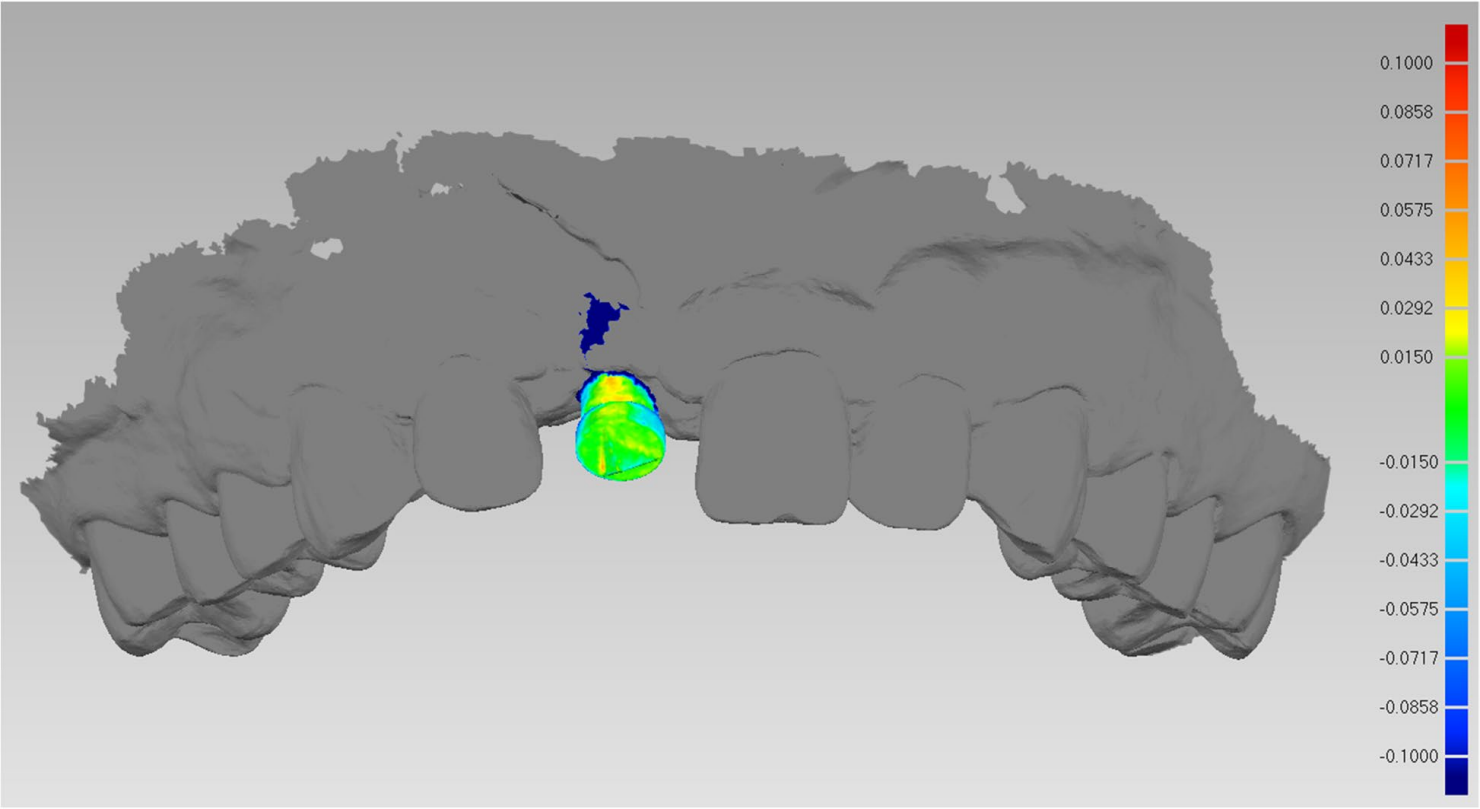
The spectrum illustrated the registration accuracy when aligning the “STL-IOS” with the “STL-Registration unit” to generate the “STL-IOS with implant”

When aligning the “STL-IOS” with the “STL-Registration unit” to generate the “STL-IOS with implant”, the registration error obtained by calculating the mRMS in 48 implants is 0.014 ± 0.027 mm. The spectrum also illustrated the registration accuracy (Fig. 12).

**Fig. 13:**
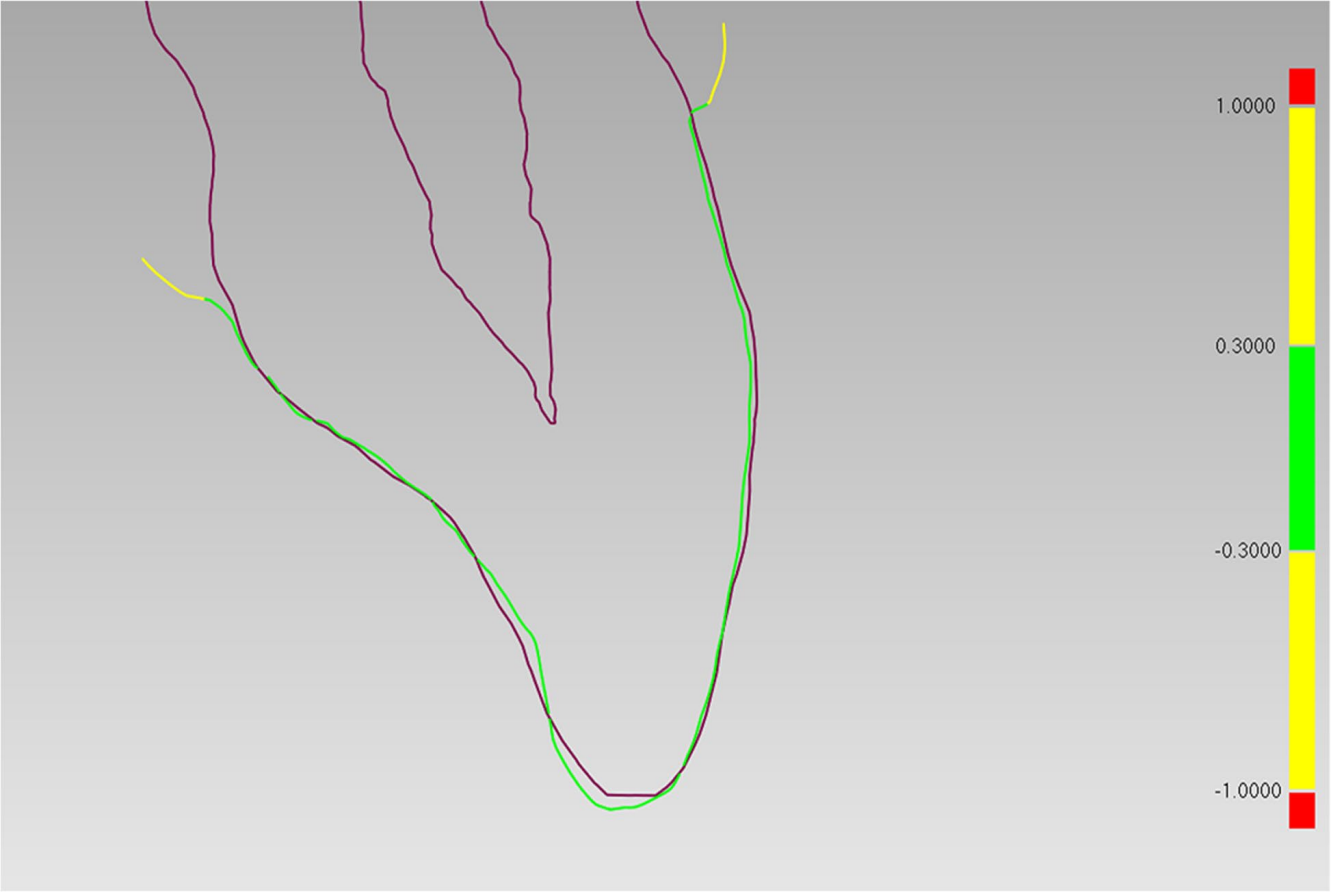
On the cross-sectional plane determined by the long axis and the gingival zenith of the adjacent tooth, the purple line segments represented the contour of the adjacent teeth obtained from CBCT scans. While the other line represented the contour of the crown obtained from IOS. Since the 2D deviation of corresponding points all fell within ± 0.3 mm, the crown contour was in green

At the 12 measurement points on the 4 adjacent teeth of patients in 2 groups, the average registration deviation between the CBCT and IOS were listed on Table 1. The overall deviation values ranged from 0.14 ± 0.05 mm to 0.21 ± 0.08 mm. The average deviation at the midpoint of incisal edge was 0.22 ± 0.08 mm for single implant group and 0.18 ± 0.06 mm for dual implant group (*p* = 0.22). The average deviation at the apex of the lingual tubercle was 0.18 ± 0.06 mm for single implant group and 0.19 ± 0.10 mm for dual implant group (*p* = 0.70). The average deviation at the labial prominence was 0.14 ± 0.05 mm for both groups (*p* = 0.78). No statistically significant differences were found between the two groups. The 2D cross-sectional images of the adjacent teeth in the implant area demonstrated promising alignment between CBCT and IOS (Fig. 13).

**Table 1:** The linear deviations of the registration procedure between the IOS and CBCT data

Group	Deviation values (Mean ± SD mm)		
	At the midpoint of incisal edge	At the apex of the lingual tubercle	At the labial prominence
Single Implant	0.22 ± 0.08	0.18 ± 0.06	0.14 ± 0.05
Dual Implant	0.18 ± 0.06	0.19 ± 0.10	0.14 ± 0.05
Total	0.21 ± 0.08	0.19 ± 0.07	0.14 ± 0.05

In the single implant group, the linear deviation at the midpoint of incisal edge were measured in 24 patients with 96 tooth sites. The mean values and SD of 96 data were calculated. In the same manner, the linear deviations at 3 types of reference points in the two groups were recorded and calculated

*SD* Standard deviation

**Fig. 14:**
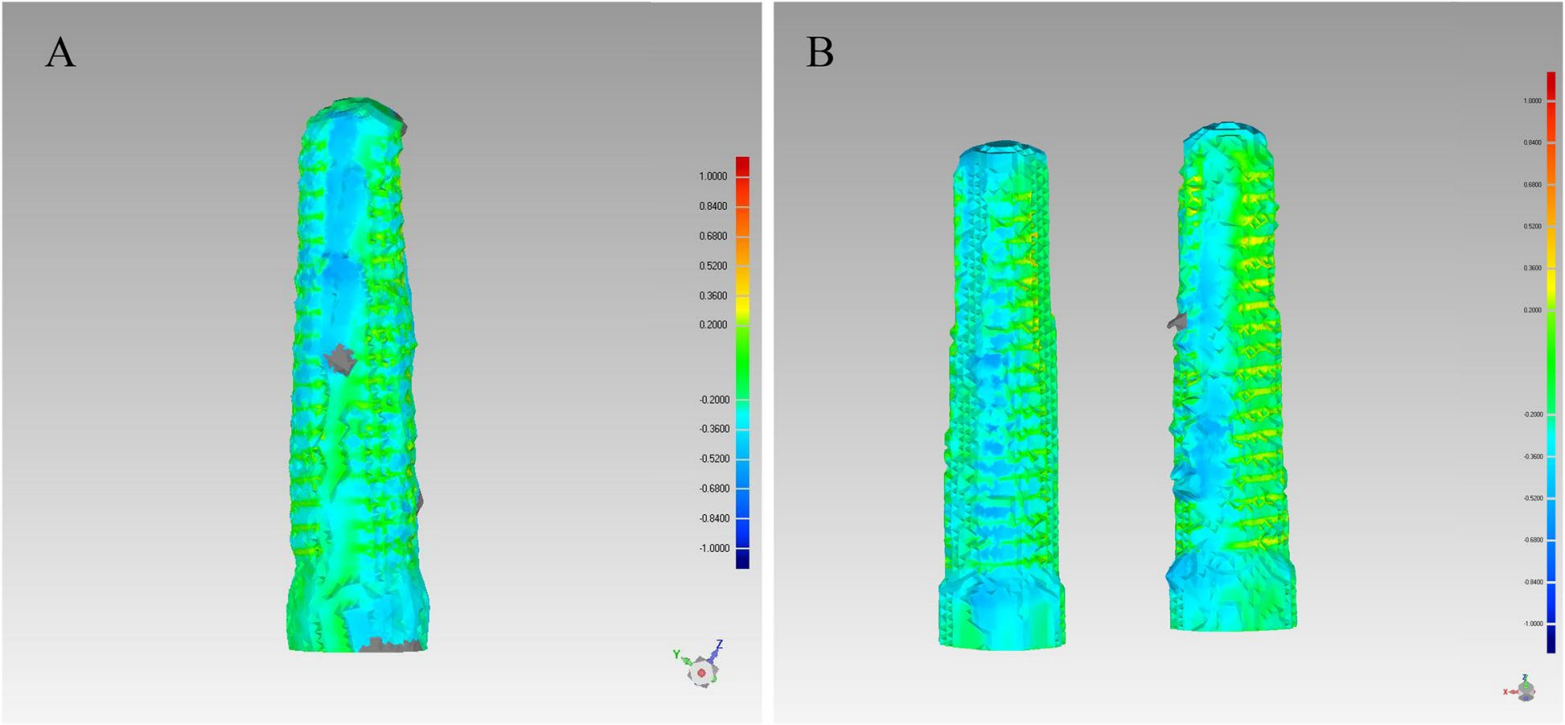
The spectrum presented the 3D deviations between the two methods. **A** single implant group;** B** dual implant group

**Fig. 15:**
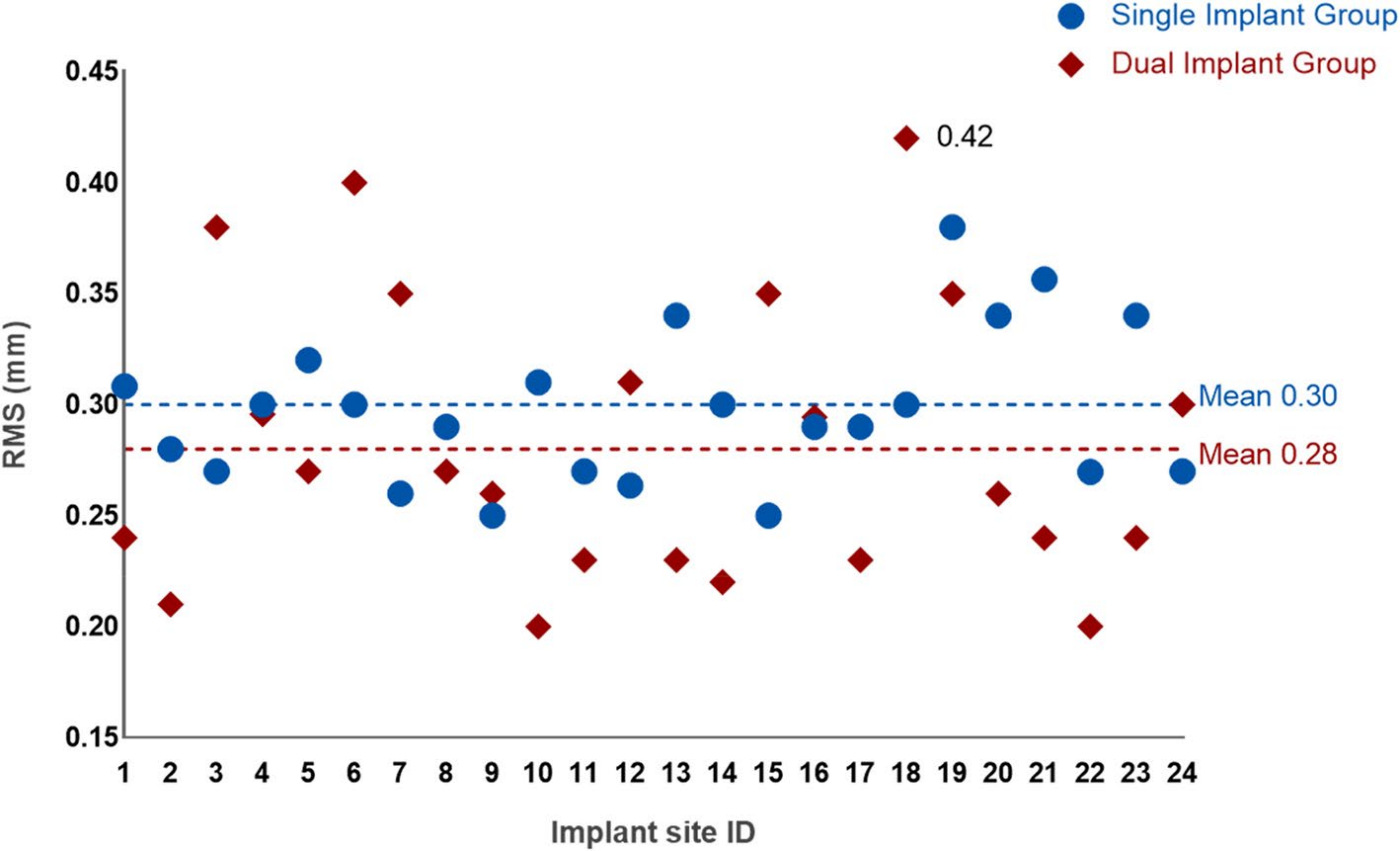
The scatter plot depicted the RMS values of all superimposed implants (*n* = 48) in the two groups

The spectrum presented the 3D deviations between the two methods (Fig. 14). The mean Root Mean Square (mRMS) for all superimposed implants (*n* = 48) was 0.29 ± 0.05 mm. The mRMS value was 0.30 ± 0.03 mm in the single implant group and 0.28 ± 0.06 mm in the dual implant group. No statistically significant difference was found in mRMS values between the two groups (*p* = 0.27). Table 2 showed the number of patients and implants as well as the mRMS results. All RMS values were displayed in Fig. 15.

**Table 2:** The mRMS values of the superimposed implants obtained by CBCT and DRM

	Total	Single implant group	Dual implants group
Patients	36	24	12
The number of implant sites	48	24	24
mRMS (Mean ± SD mm)	0.29 ± 0.05	0.30 ± 0.03	0.28 ± 0.06

*SD* Standard deviation

The outcomes showed an overall superimposed implants mRMS of 0.29 ± 0.05 mm (CI = 0.27 to 0.30), indicating that the majority of samples had registration deviations that were predominantly distributed in the region of less than or equal to 0.30 mm.

## Discussion

The purpose of this clinical study was to verify the accuracy of the DRM in obtaining implant positions. The results of this study showed an overall average deviation of 0.29 ± 0.05 mm between the implant positions obtained via the DRM and CBCT scan method. There was no significant difference between the deviations of single and dual implant groups using the DRM. This study indicated that the DRM can be used to evaluate implant position and its relative position to the surrounding structures in single and dual implant treatment, with an acceptable deviation range in comparison with the postoperative CBCT scan.

We chose the maxillary incisors as the focus for assessing the accuracy of the DRM for two primary reasons. 1) Previous studies have consistently demonstrated that the IOS accomplishes the scanning of the target area based on the image stitching principle of feature point recognition [27, 28]. The accuracy of the IOS is influenced by the scanning range and the anatomical features of the scanned objects [27]. The smaller the scanning range and the fewer anatomical feature points of the scanned object, the lower the scanning accuracy [29, 30]. Given that scanning the maxillary incisor region typically involves crossing the dental arch (two quadrants) and considering the reduced anatomical features of anterior teeth compared to the posterior teeth, this leads to lower scanning accuracy [31–33]. Therefore, to validate the applicability of the DRM, we specifically included the maxillary incisors characterized by relatively lower scanning accuracy. If the accuracy of the DRM is confirmed to be within clinically acceptable ranges for determining the position of anterior dental implants, it suggests that the method's accuracy in obtaining implant positions in a single quadrant or the posterior region, where the IOS accuracy is higher, also falls within clinically acceptable ranges. 2) The purpose of the study was to evaluate the accuracy of the DRM for implant positioning and compare it with the CBCT-based method, which required the inclusion of cases where postoperative CBCT was taken. From an ethical standpoint, exposing patients to additional radiographic examinations for research purposes is not warranted. For post-surgical examination of the implant surgeries without bone grafting or complications, CBCT seems inappropriate due to increased radiation exposure, violating the “As Low as Diagnostically Acceptable being Indication-oriented and Patient-specific (ALADAIP)” principle [2]. Therefore, we did not include cases of simple implantation of posterior teeth without bone augmentation to avoid subjecting them to postoperative CBCT scans.

However, examining the bucco-palatal position of the implants in the anterior maxilla and their relationship with the labial bone plate is essential. Moreover, patients requiring implant treatment in the anterior maxillary region, especially the maxillary incisor area, usually involve bone augmentation surgery. Therefore, pre- and post-operative CBCT scans are necessary procedures. Evaluating the DRM using CBCT data from this subgroup of patients is appropriate. To clarify, the CBCT data in this study originated from essential scans conducted during the implant treatment procedures rather than for research purposes.

The DRM was used to investigate the implant positions in four steps. First, an IOS was taken after connecting the ISB with the inserted implant. Second, a virtual registration unit was constructed through a reverse engineering process. The registration unit consisted of an implant replica and a compatible ISB. Third, the relative position between the postoperative implant and the adjacent dentition was identified through the first registration, which was performed based on the scan body as the reference point. Fourth, if the DRM is used to examine the postoperative positions of implants, a second registration should be performed. Clinicians can superimpose the first registration data and the preoperative CBCT volume data to enable visualization of the 3D position of the inserted implant and its relationships with the surrounding anatomical structures.

To evaluate the accuracy of the DRM, we superimposed the implant positions obtained using this method with those obtained from postoperative CBCT scans. By extracting the two types of implant positions exclusively, the RMS value was calculated to evaluate the deviation in 3D perspective. To assess the accuracy of registration in data processing, three types of reference points in the adjacent tooth area were selected and the deviation in a 2D cross-sectional plane was recorded. Additionally, the average values of the deviation values were calculated, which represent the overall performance and reliability of the registration technique.

In the traditional implantation surgery process, patients have to leave the operating room and go to the radiology department for radiographic examination. However, the use of 2D radiographs, such as periapical and panoramic radiographs, is limited in evaluating the implants from a 3D perspective, and the images can be subject to torsion, deformation, and amplification. Although CBCT examination provides a 3D perspective, the radiation exposure is much higher than that of 2D radiographs. Furthermore, it should be noted that the quality of CBCT images can be affected not only by the presence of brackets and restorations [2, 7, 34, 35], but also by all dense objects like dental enamel and titanium implant itself, which can give rise to artifacts as a result of the beam hardening effect to a lesser extent, making it unclear for evaluation of implant with adjacent anatomy [36, 37]. In contrast, the DRM can determine the 3D position of the postoperative implant without radiation exposure. This method avoids the issues of image torsion, deformation, and artifacts caused by traditional radiographic examinations, and reduces cost for patients [13].

If a postoperative radiographic examination shows that the implant is not optimally positioned, both the clinician and the patient will need to invest more time and effort to adjust the location to achieve the desired surgical outcomes. However, this DRM provides a solution. Following the implant's placement, an IOS is conducted, and the facial data of the scan is registered with the preoperatively obtained registration unit. This registration utilizes the ISB as a reference point to determine the implant's relative position with the dental arch. The registered data is then overlaid onto the preoperative CBCT data. This technique instantly provides feedback on the 3D position of the implant within the jawbone in the operating room. Due to the voxel size of CBCT being 0.20 mm, it is not possible to achieve registration accuracy smaller than 0.20 mm when aligning data derived from CBCT [35, 37]. Nevertheless, the lab scanner's manufacturer (3Shape E4, 3Shape, ISO 12836) reports a high level of accuracy at 4 μm. In a clinical study that assessed the precision of single and multiple implant scans, various intraoral scanners including CS 3600®, Trios3®, DWIO®, Omnicam® and Emerald® were evaluated. The average accuracy of single-implant scans from these scanners was approximately 30 μm. In case of two-implant scans, while there was a slight reduction in accuracy, it remained within 60 μm [38]. As a result, the DRM offers better precision compared to the CBCT method.

After aligning the implant positions obtained through the DRM with those obtained through CBCT, the average RMS deviation between the two implant positions was found to be 0.29 ± 0.05 mm, with a maximum RMS deviation of 0.42 mm. The first step registration deviation between the registration unit and IOS was measured to be 0.014 ± 0.027 mm, as indicated by the mRMS value. The second step registration deviation between CBCT and IOS was observed to be within 0.15 mm—0.25 mm, determined by measuring the linear deviation on the reference points in the adjacent teeth. Given the CBCT's voxel size of 0.20 mm and considering the registration accuracy of the two steps (0.014 ± 0.027 mm, and 0.15 mm—0.25 mm), the mRMS value (0.29 ± 0.05 mm) was constrained by these factors, theoretically yielding results greater than 0.20 mm—0.30 mm.

According to the available literature, a significant variation in errors has been observed when making linear measurements on CBCT images. Therefore, when using CBCT, it is recommended to take into account a 2 mm safety margin to ensure adequate space from adjacent anatomical structures [35]. Thus, when the average deviation of the digital method is below this safety distance, it accurately depicts the spatial relationship between the implant and the surrounding anatomical structure.

Previous studies did not use RMS to assess differences in implant positions obtained from two different methods. For example, an clinical study conducted by Zhou et al. [10] showed that the average linear deviation of the DRM was within 0.3 mm, but this was calculated based on the distance between the entry point and apex point of the implants obtained by the two methods. In another in vivo experiment conducted by Tang et al. [11], the accuracy of the digital registration technique for implant positioning was assessed. Their study differs from the present research in several aspects. First, the postoperative optical scanning step scanned a study cast rather than the dentition. Second, the number of implants was not specified. Third, the contour of the implant was processed as a simulated cylinder, which might lead to errors. Fourth, the study also used entry point, apex point and axis as parameters to evaluate the difference in implant position. The aforementioned two studies used the linear distance deviation at the entry or apex points between two implants, as well as angular deviation of the axes, to evaluate the consistency of two implant positions. However, this approach may lead to some issues. Firstly, these three parameters were frequently used to evaluate the precision of implant surgical guides. Secondly, manually selecting entry and apex points in linear or angular measurements often introduced errors when the two implant positions were very close. To avoid these issues, manual point selection was not employed in our study, and RMS was used to assess two strongly proximate implants to determine the average difference in a 3D perspective.

This study evaluated the efficacy of the DRM for single and double implant registration and found that the results were similar. In previous literature that investigated the accuracy of the DRM [10, 11, 39–41], no specific studies were found comparing the impact of the number of implants on the accuracy of this method. The results of this study demonstrated that the method could be used in single and dual implant treatments to assess the implant's position.

The registration accuracy between IOS and CBCT data is a critical factor that impacts the accuracy of the DRM. In this study, the registration accuracy of IOS and CBCT data was evaluated by measuring the linear deviations of reference points on four adjacent tooth positions with implants. When performing the superimposition of “STL-IOS with implant” and “STL-CBCT”, the linear deviation measurement in the adjacent teeth on a typical 2D cross-sectional plane can provide a straightforward indication of the registration accuracy. Three reference points were manually selected for each tooth, and labial prominence showed the highest registration accuracy, with deviations measuring less than 0.16 mm. This was followed by the apex of the lingual tubercle. The midpoint of the incisal edge exhibited the lowest accuracy, although its average deviations still fell within the range of 0.19–0.21 mm. In this study, the registration deviation between CBCT and IOS data was found to be similar to the results reported in prior research, demonstrating an average deviation of approximately 0.2 mm [24].

In this clinical study, certain factors such as the aforementioned artifacts in CBCT images could potentially compromise the accuracy of the experimental results. It's reported that even light metals like titanium can cause significant beam hardening artifacts [42]. These artifacts appear as dark streaks during the 3D reconstruction process [43], which can render anatomical structures ambiguous and greatly diminish the contrast between adjacent regions [34]. To ensure the most reliable outcomes, some efforts were made to reduce or eliminate the effect of such artifacts. After CBCT scans, shadows and streaks caused by implants were removed in the software (Planmeca Romexis; Planmeca Oy, Finland). However, there still exists a possibility of minor remaining artifacts, which may potentially impact the outcomes of the experiment. The DRM, on the other hand, is not affected by artifacts, which demonstrates its superiority over CBCT in obtaining implant positions.

The study has some limitations. Firstly, the assessment of the DRM in this study solely focused on the accuracy of obtaining the positions of single and dual implants. It did not address the accuracy of acquiring positions for multiple implants, which necessitates further investigation in future studies. Secondly, the number of implants included in this study was relatively small. The accuracy of this method requires further validation in larger clinical studies with a more substantial sample size.

Additionally, there are certain limitations to the clinical application scenarios of DRM. The accuracy and feasibility of the DRM were also influenced by the number and location of missing teeth, as well as the complexity of the surgical procedure. When there were more missing teeth or they were located in multiple quadrants, the accuracy of the DRM may be compromised due to the lack of fixed reference points [38, 44, 45]. Therefore, in such cases, the DRM is not currently feasible. Further studies are needed to determine whether alignment reference point such as fixation pins and temporary implants could be used for the DRM.

It is also important to note that the implant position obtained by the DRM is deduced and does not represent the actual implant position. Therefore, it cannot be applied in implant surgeries where the original bone contour changes. For complex implantation procedures, such as guided bone regeneration (GBR) and maxillary sinus floor elevation, it is still recommended to use CBCT for examination. However, in cases where immediate implantation only involves bone grafting in the jumping gap without changing the original bone contour, the DRM can be used to locate the implant.

## Conclusion

First, when obtaining 3D positions of implants, the DRM exhibited a maximum deviation of less than 0.50 mm and an average deviation of 0.29 ± 0.05 mm when compared to CBCT scans. These deviations were within the clinically acceptable limits, indicating that the DRM was a reliable tool for accurately assessing implant positions.

Second, no significant difference was found in the accuracy of 3D positioning between single and dual implant using the DRM. This suggests that the method was equally effective for both single and dual implant treatments.

Third, the results of adjacent tooth registration indicate that the DRM may effectively control the alignment errors during the data processing procedure.

## Data Availability

All essential data is presented in the manuscript. The step-by-step datasets and images during the current research are available from the corresponding author on reasonable request.

## Abbreviations

DRM: Digital Registration Method
CBCT: Cone-Beam Computed Tomography
3D: Three-Dimensional
2D: Two-Dimensional
IOS: Intraoral Scan
ISB: Intraoral Scan Body
STL: Standard Tessellation Language
FOV: Field-of-View
DICOM: Digital Imaging and Communications in Medicine
RMS: Root Mean Square
mRMS: Mean Root Mean Square
SD: Standard Deviation
